# Calibration and accuracy of the geno2pheno co-receptor algorithm for predicting HIV tropism for single and triplicate measurements of V3 genotype

**DOI:** 10.1186/1758-2652-13-S4-O8

**Published:** 2010-11-08

**Authors:** LC Swenson, D Knapp, PR Harrigan

**Affiliations:** 1 BC Centre for Excellence in HIV/AIDS, Vancouver, Canada

## Background

The geno2pheno algorithm (g2p) can give dichotomous tropism based on a selectable "false positive rate" (FPR), reflecting the proportion of individuals inappropriately called "non-R5" and falsely excluded from taking CCR5 antagonists. The effect of replicate genotype measures and different FPR values remains controversial. Here we characterize different FPR "cut-points" in predicting tropism for single vs multiple replicates for interpreting V3 genotype based on data from the clinical trials of Maraviroc (MVC) in experienced patients.

## Methods

The first study population comprised all patients screened for MOTIVATE 1 (N=1399; 44% non-R5 by original Trofile) for whom both triplicate and single V3 genotypes were available. We also examined virological response (defined as a week 8 decrease ≥2 logs and/or to <50 copies/ml) in an outcome dataset of 547 patients who received MVC+optimized background therapy in the MOTIVATE-1, 2 or A4001029 studies with very limited background antiviral activity from other agents (wSS <1).

## Results

Triplicate sequence analyses typically identified 10-25% more individuals with non-R5 virus compared to single replicates. A comparison of the predicted FPR by g2p to the virologically defined FPR at different g2p cut-points showed an excellent correlation (r2 =0.99; see Table 1), but appeared to be calibrated conservatively (slope =1.5 for single assays or 1.7 for triplicate assays). Some of this miscalibration likely reflects a contribution from background therapies. For comparison, the FPR of Trofile in this population was 3.9% (N=49 DM patients).

**Table:** 

MOTIVATE-1 Screening (N=1399)			Virological Outcome Set (N-=47)		
	**Number non-R5**		**Number Non-Rf**		**Actual FPR**	

**G2p FPR**	**(single)**	**(triplicate)**	**(single)**	**(triplicate)**	**(single)**	**(triplicate)**

1	100	114	6	9	0.6	0.6

2	241	288	25	36	1.3	1.3

3	303	368	39	47	2.3	2.6

4	362	427	48	57	3.6	4.2

5	396	459	52	64	4.5	5.2

**5.75**	**423**	**486**	**57**	**71**	**6.1**	**7.4**

6	433	496	62	77	6.8	8.4

7	476	533	71	89	8.4	10.0

8	507	563	78	98	9/7	11.7

9	548	605	88	114	12.0	13.9

**10**	**562**	**620**	**89**	**116**	**12.3**	**13.9**

15	646	715	132	161	21.4	23.6

**20**	**742**	**805**	**174**	**205**	**28.5**	**32.4**

## Conclusions

The g2P algorithm shows the expected association with observed virological response, but this testing procedure may be more conservative than expected from the nominal FPR values, particularly for triplicate sequence analysis. A g2p FPR value above 10 likely excludes too many individuals who could respond to therapy if this cut-off is employed to screen individuals for maraviroc.

